# Outcome of Cerebellar Injury with Intraventricular Hemorrhage

**DOI:** 10.15844/pedneurbriefs-29-2-3

**Published:** 2015-02

**Authors:** Charu Venkatesan

**Affiliations:** 1 Division of Neurology, Department of Pediatrics, Cincinnati Children's Hospital Medical Center, Cincinnati, OH

**Keywords:** Cerebellar injury, Cerebral palsy, Hydrocephalus, Intraventricular hemorrhage, Outcome, Preterm

## Abstract

Investigators from the Department of Pediatric Neurology, Morinomiya Hospital, Osaka, Japan performed a retrospective IRB approved study of the prevalence of cerebellar injury (CI) and effect on functional outcomes among preterm children with intraventricular hemorrhage (IVH) and cerebral palsy (CP), comparing them to infants with post-hemorrhagic hydrocephalus (PH).

Investigators from the Department of Pediatric Neurology, Morinomiya Hospital, Osaka, Japan performed a retrospective IRB approved study of the prevalence of cerebellar injury (CI) and effect on functional outcomes among preterm children with intraventricular hemorrhage (IVH) and cerebral palsy (CP), comparing them to infants with post-hemorrhagic hydrocephalus (PH). 69 children born between gestational ages 22 and 34 weeks (40 males and 29 females, between 6 and 13 years of age) were studied. Patients were divided into four groups (PH+/CI+, PH+/CI−, PH−/CI+, and PH−/CI−) according to the presence or absence of PH or CI. Outcome measures included CP, ability to walk, ability to communicate verbally, the complication of epilepsy, and the presence of severe visual impairment.

In this study, the prevalence of cerebellar injury in preterm infants with CP after perinatal IVH was 58%. The proportion of those with impaired ambulatory ability, speech production and epilepsy was significantly higher in the PH+/CI+ and PH−/CI+ groups compared to the PH−/CI− and PH+/CI− groups. Severe visual impairment was found only in the PH+/CI+ and the PH−/CI+ groups. The findings of this study suggest that cerebellar injury worsens motor and verbal function as well as epilepsy, and its influence is even greater than that of PH. [1]

COMMENTARY. The cerebellum undergoes a rapid rate of growth from 24 to 40 weeks gestation, increasing 3 to 5-fold in volume during this time period [2]. Acquired cerebellar abnormalities in the premature infant are caused by destructive (e.g. hemorrhagic or ischemic injury) or impaired development [2, 3]. Mechanisms for impaired development have not been identified but there is an association between this entity and supratentorial lesions such as periventricular leukomalacia and IVH [2]. MRI imaging has shown that the incidence of cerebellar injury is more common than previously suggested by ultrasound. Johnson et al., found that 45% of children born with extremely low birth weight and who had cerebral palsy had cerebellar injury detected by MRI [4]. Lesions are also dependent on the degree of prematurity; for example, one study found that cerebellar hemorrhage has an incidence of 15% in infants weighing less than 750 g compared to about 2% in infants weighing 1000 – 1499 grams [5]. Other studies, including by Zayek et al., have also found impairments in motor and cognitive function in premature infants with cerebellar injury [6]. However, the size of the lesion may also impact neurodevelopmental outcome. Tan et al. found that preterm infants with smaller hemorrhages (1-3 mm) did not have cognitive impairment on developmental testing performed at 3-6 years of age [7]. These studies highlight the complexity of cerebellar injury in the preterm infant. Cerebellar injury can affect not only motor function but also cognitive, affective and social function. These infants require close vigilance with intervention for neurodevelopmental complications and epilepsy.
